# Comparison of plasma pTau217 assays on different platforms

**DOI:** 10.1002/alz70856_102192

**Published:** 2025-12-25

**Authors:** Hongming Zhang, Jingwen EH Tan, Nan Zhang, John Zhongping Lin

**Affiliations:** ^1^ Frontage Laboratories, Exton, PA, USA; ^2^ Frontage, EXTON, PA, USA

## Abstract

**Background:**

Plasma pTau217 was recently discovered as a prominent blood‐based biomarker in the early diagnosis of Alzheimer's disease in which it can discriminatively predict AD development decades ahead of symptom onset. Numerous pTau217 assays have been developed on different platforms (1‐4). To compare the platform performing features, we selected 7 of them and did the head‐to‐head comparison of the pTau217 assays that have been comprehensively or newly explored in the immunoassays in clinical trials and/or LDT diagnostic applications.

**Method:**

Plasma pTau217 assays were performed following the respective manufacturer instructions of the assays/platforms with critical reagents provided in the respective kits from the manufacturers: pTau217 on Bechman Coulter DxI 9000 Access Immunoassay Analyzer, pTau217 on Fujirebio Lumipulse G1200, pTau217 on AstrBio Ast‐Sc‐Semi, ALZpath pTau217 on Quanterix Simoa HD‐X, S‐plex pTau217 on MSD Sector S600, pTau217 on Sigma SMCxPRO, and CNS Panel 120 on Alamar ARGO HT.

**Result:**

We evaluated the performance of human plasma pTau217 assays on the 7 selected platforms. The following parameters were evaluated for their performance: instrument hardware and software design and operational performance, sample preparation, automation and throughput, total run time from sample preparation to run data generation, accuracy and precision, detection range, sensitivity and detectability, correlation with other diagnostic methods, cost per run, and convenience of maintenance. The results demonstrated that each of the instruments has its unique features for plasma pTau217 analysis.

**Conclusion:**

Overall, the 7 tested pTau217 assays on the respective platforms showed good performance with their unique features. The data will provide a very useful reference for users in choosing suitable platforms for their clinical research or LDT diagnostic applications.

